# Cortisol and testosterone increase financial risk taking and may destabilize markets

**DOI:** 10.1038/srep11206

**Published:** 2015-07-02

**Authors:** Carlos Cueva, R. Edward Roberts, Tom Spencer, Nisha Rani, Michelle Tempest, Philippe N. Tobler, Joe Herbert, Aldo Rustichini

**Affiliations:** 1 Departamento de Fundamentos del Análisis Económico, Universidad de Alicante, Spain; 2 Division of Brain Sciences, Department of Medicine, Imperial College London, UK; 3 Department of Psychiatry, University of Cambridge, UK; 4 Cambridgeshire and Peterborough NHS Foundation Trust, Elizabeth House, Fulbourn Hospital, Cambridge, UK; 5 Royal College of Psychiatrists, UK; 6 Laboratory for Social and Neural Systems Research, Department of Economics, University of Zurich, Switzerland; 7 John van Geest Centre for Brain Repair, Department of Clinical Neurosciences, University of Cambridge, UK; 8 Department of Economics, University of Minnesota, USA; 9 Department of Physiology, Development and Neuroscience, University of Cambridge, UK

## Abstract

It is widely known that financial markets can become dangerously unstable, yet it is unclear why. Recent research has highlighted the possibility that endogenous hormones, in particular testosterone and cortisol, may critically influence traders’ financial decision making. Here we show that cortisol, a hormone that modulates the response to physical or psychological stress, predicts instability in financial markets. Specifically, we recorded salivary levels of cortisol and testosterone in people participating in an experimental asset market (N = 142) and found that individual and aggregate levels of endogenous cortisol predict subsequent risk-taking and price instability. We then administered either cortisol (single oral dose of 100 mg hydrocortisone, N = 34) or testosterone (three doses of 10 g transdermal 1% testosterone gel over 48 hours, N = 41) to young males before they played an asset trading game. We found that both cortisol and testosterone shifted investment towards riskier assets. Cortisol appears to affect risk preferences directly, whereas testosterone operates by inducing increased optimism about future price changes. Our results suggest that changes in both cortisol and testosterone could play a destabilizing role in financial markets through increased risk taking behaviour, acting via different behavioural pathways.

Numerous reasons have been proposed to explain why financial markets undergo periods of instability. These include: debt accumulation^1^, incorrect beliefs about the earnings process^2^, limits to arbitrage^3^, asset incompleteness^4^, herding^5^,^6^ or momentum trading^7^,^8^. Yet influential economists still recognize the key role played by the unpredictability of human motivation^9^. John Maynard Keynes captured this idea originally with the term ‘animal spirits’: “a spontaneous urge to action” ultimately responsible for our decisions to take risks impulsively rather than after a process of careful calculation^10^. Alan Greenspan and Robert Shiller later used the phrase ‘irrational exuberance’ to describe a possible cause of overvaluations in asset markets^11^,^12^. However, despite the prominence of this idea, the physiological basis for “irrational exuberance” has only recently begun to be explored^13^.

Trading floors are highly stressful and competitive environments. In both humans and non-human animals, such conditions are known to be associated with fluctuations primarily in two endogenous steroid hormones: cortisol and testosterone. Cortisol is elevated in response to physical or psychological stress^14^, and is particularly sensitive to situations of novelty, uncertainty or threat^15^. Acute increases in cortisol promote fear, physical arousal and sensation seeking^14^. Testosterone has been found to both predict success rates and confidence in competitive encounters with levels increasing in response to victories^16^,^17^ or challenging situations, thought to be part of a positive feedback loop termed the ‘winner effect’^18^,^19^. Testosterone has also been closely linked with perceived social status^20^,^21^,^22^,^23^. In men, elevated levels of testosterone have been associated with increased aggression, sexual function and mood^24^,^25^,^26^. Thus, the evidence would seem to indicate that either hormone could play a role in modulating individual preferences for risk taking and market instability, particularly when participating in an arena as stressful and competitive as a modern financial market.

This possibility is supported by data from field investigations examining the hormone levels of professional traders. One study reported that traders made significantly higher profits on days when their morning testosterone levels were above their daily average, and that increased variability in profits and uncertainty in the market was reliably associated with elevations in their cortisol levels^27^. A second study found that traders’ second-to-fourth digit ratio – a postulated indicator of pre-natal exposure to testosterone^28^–was also associated with higher profits and career longevity^29^.

If altered levels of either hormone were to affect the appetite for financial risk, could this in turn destabilize the market as a whole? Since the fundamental value of an asset in a financial market is an aggregation of the stochastic stream of future dividends, trading at prices higher than the fundamental value is only profitable when there is a widespread belief that other traders will continue to buy at prices *even further* away from fundamental values. Such speculative and ultimately unsustainable trading strategies are risky, and critically contribute towards price instability. An increased willingness to take risks makes these uncertain investments, everything else being equal, more desirable, and this in turn makes price bubbles and financial market instability more likely.

However, direct evidence to support a link between hormones and investment behaviour is limited^30^,^31^,^32^,^33^, and it is not clear whether any of these findings can be generalized to trading in financial markets, where other factors such as confidence and ability are likely to play an important role^34^. Most importantly, none of these investigations provide an answer to the more economically significant question of aggregate market effects. Thus, the conjecture that endogenous variations in either hormone could destabilize financial markets remains unaddressed.

Here we first tested the hypothesis that endogenous levels of either cortisol or testosterone would predict risk taking and price instability in a well-understood experimental trading environment that mimics the key features of a real-world financial market. This experiment involved no hormone administration. Changes in subjects’ hormonal levels could only be induced by the natural reaction to our experimental trading environment. In two additional experiments with young males, we induced changes in either hormone by administering cortisol or testosterone. This allowed us to test whether increased levels of either hormone affected performance in an individual investment game. Specifically, we were interested in whether elevated levels of testosterone or cortisol increased preferences for investing in risky rather than safe assets.

## Results

### Associations between endogenous hormones in an experimental asset market

**Figure 1:**
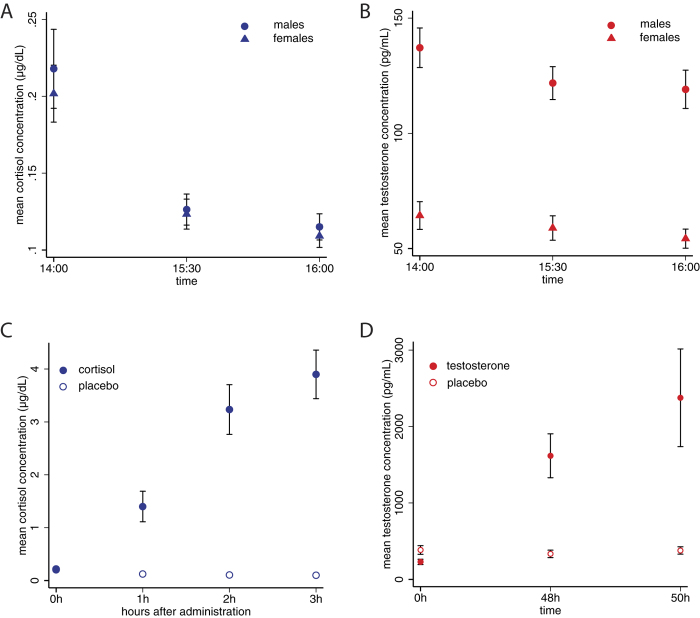
Salivary hormone concentrations. Error bars, mean ± SE. (**A**) Asset market experiment. Mean salivary cortisol concentrations in men and women immediately before the asset market (14:00), immediately after the asset market (15:30) and half an hour after (16:00), N = 420. (**B**) Asset market experiment. Mean salivary testosterone concentrations in men and women immediately before the asset market (14:00), immediately after the asset market (15:30) and half an hour after (16:00), N = 412. (**C**) Cortisol administration experiment. Mean salivary cortisol concentrations in placebo and cortisol treatments (N = 200). The drug or placebo administration occurred immediately after the baseline sample at 0 h, and the experimental task was performed after the second sample at 1 h. (**D**) Testosterone administration experiment. Mean salivary testosterone concentrations in placebo and testosterone treatments at baseline (0 h), before (48 h) and after (50 h) performance of the trading task (N = 173).

We investigated whether naturally occurring variations in either endogenous cortisol or testosterone levels predict individual differences in trading behaviour and aggregate price stability in real multi-player markets using an asset market experiment with real monetary incentives. Male, female or mixed groups of participants traded amongst themselves, and salivary levels of cortisol and testosterone were measured before and after each trading session (see Fig. 1a,b).

We employed an experimental design that has been used previously to analyse stock market bubbles in the laboratory^35^, developed from an earlier paradigm^36^,^37^,^38^. A group of typically 10 subjects traded cash and assets in a computerized bilateral exchange–a double-auction. Markets consisted of 15 trading periods, each lasting 2 minutes. After each trading period, the assets yielded a random positive or negative dividend drawn from a known distribution with zero expected value. At the end of the final trading period, each asset paid a maturity value of 1 GBP. Subjects entered the market with 10 units of the asset and a cash loan of ≈28 GBP (details in supplementary material).

This experimental paradigm implements the main characteristics of actual financial markets in which several participants trade stocks as buyers and sellers and determine prices freely in a sequence of bilateral exchanges. In these markets, the behaviour of each trader is typically affected by the behaviour of the other traders. This is particularly important in situations of price instability, such as during bubbles or crashes, where initial price movements can become exaggerated due to herd–like behaviour and momentum trading^39^. Therefore, to assess whether individuals with high endogenous cortisol or testosterone might trigger market instability, we focused on the first trading period, since here subjects had minimal information about the behaviour of other traders. We then analysed correlations between individual hormone levels before and after trading with behaviour throughout the market. Finally, we examined the relationship between average hormonal levels in the market and aggregate price stability.

The fundamental value of a stock in our markets – the expected total dividend payout – was 1 GBP. Therefore, prices should not deviate substantially from 1 GBP in markets with rational traders. Furthermore, since there are no gains from trade, these markets should exhibit very few transactions in equilibrium. Any purchases of stock substantially above this price or sales substantially below this price constitute mispricing as they do not reflect the fundamental stock value, to which the market tends to return in the long run. Since transactions at prices away from the fundamental value can only be profitable if the market moves even further away from it in the future, these trading strategies carry a high risk and induce market instability.

### Associations between endogenous hormone levels and individual trading behaviour

Given the structure of our asset market, traders who accept or submit aggressive bids (high buying prices) or asks (low selling prices) more frequently will execute a higher number of transactions because their bids and asks will be preferentially selected by other traders. Thus, the number of transactions is a good indicator of the degree of risk or aggressiveness of a trader’s strategy. We regressed *trading activity* on pre-auction cortisol, testosterone, and a cortisol-testosterone interaction separately for men and women, including dummy variables for each market (see supplementary table 1a). Cortisol was strongly associated with greater trading activity in men (*t* = 4.35, *P* = 0.001, *R*^*2*^ = 0.381), whereas testosterone correlated negatively but not significantly with trading activity in the same regression (*P* = 0.1, *R*^*2*^ = 0.381, also see Fig. S1). We found no evidence for a significant interaction between cortisol and testosterone, as is proposed by the dual-hormone hypothesis^40^. In contrast to the behaviour of men in the experiment, women exhibited a borderline significant negative correlation between trading activity and cortisol (*P* = 0.08, *R*^*2*^ = 0.326), and a positive insignificant correlation with testosterone (*P* > 0.6, *R*^*2*^ = 0.326). Pooling together male and female data into a single regression (*R*^*2*^ = 0.268), we found a significant positive effect of *male* (*t* = 2.84, *P* = 0.01) and of the interaction *male-cortisol* (*t* = 2.64, *P* = 0.02), and a significant negative interaction *male-testosterone* (*t* = −2.89, *P* = 0.01). Note, however, that unlike for cortisol, we did not find a significant effect of testosterone when analysing male and female trading activity separately, therefore this interaction may be influenced by the marked differences in testosterone levels between men and women.

To assess the degree to which transactions deviated from the fundamental stock value we constructed the variable *mispricing*. This variable corresponds to deviations of prices from fundamental value and is defined as the sum, over a subject’s purchases above the fundamental value and sales below the fundamental value in the first period (see supplementary table 1b). Using similar regressions, we estimated the variable *mispricing* as a function of individual pre-auction hormone levels (*R*^*2*^ = 0.288). Our results show a positive although insignificant correlation between men’s cortisol level and mispricing (*P* > 0.1) and a negative and insignificant correlation with testosterone (*P* > 0.2).

The results so far indicate a positive association between pre-auction endogenous cortisol and early trading activity in men but not in women. As Fig. 1a shows, cortisol levels were significantly elevated before the experiment (14:00 h) compared to samples taken at later periods (*P* < 0.01). The decline in salivary cortisol towards the end of the experiment (16:00h) is in line with the normal diurnal variation in cortisol levels^41^; however, it is also possible that pre-auction levels were unusually high because of anticipatory stress. To further check the robustness of the association between cortisol and trading behaviour we examined the correlation using data from *all* trading periods and salivary hormone measurements from both 14:00h and 16:00h. Here, cortisol at 16:00h was positively and significantly correlated with men’s trading activity (*t* = 2.37, *P* = 0.02, *R*^*2*^ = 0.195) and mispricing (*t* = 2.23, *P* = 0.03, *R*^*2*^ = 0.117), whereas cortisol at 14:00h was not (*P* > 0.3, see supplementary table 2). Thus, although pre-auction cortisol predicts early trading activity, behaviour throughout the session is correlated with cortisol levels at the end of the session.

With respect to profits, hormone levels either at 14:00h or 16:00h were not correlated with trading performance in men or in women (see supplementary table 3), suggesting that neither hormone level predicted differences in trading ability.

### Associations between endogenous hormone levels and market price instability

To investigate whether high cortisol promoted price instability in male or mixed markets, we explored whether mean *group* cortisol levels were correlated with aggregate price volatility in the market. We focused on a standard measure called *normalized absolute deviation* (*NAD*) – the sum of the deviations of prices from the fundamental value in every market transaction^35^. This measure takes into account not just prices, but trading amounts, and is therefore richer than other common measures of mispricing and volatility such as *amplitude* – the difference between the highest and the lowest transaction price observed in the market or *relative absolute deviation* (*RAD*) – the mean absolute price deviation^42^. *Amplitude* and *RAD* are insensitive to the level of activity in the market, which is commonly higher during periods of market instability and particularly during bubbles^43^. However, for completeness, we estimated the effect of average pre-auction cortisol and testosterone in the market on all three measures, controlling for whether the market was male-only, mixed or female-only.

**Figure 2:**
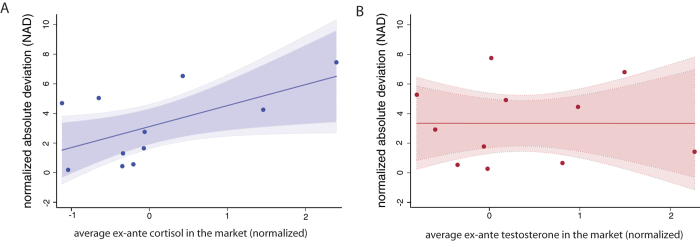
Aggregate pricing away from fundamental value and *ex-ante* average endogenous cortisol(A) and testosterone (B) in the asset market experiment. Each data point represents one market (female-only markets excluded). Line fitted from a linear regression. Shaded areas represent the 90% and 95% CI of the predicted mean. *Ex-ante* cortisol is significantly correlated with subsequent NAD in the market (linear regression with robust sandwich variance estimator, *t* = 2.63, *P* = 0.027, *R*^2^ = 0.338). Testosterone is not correlated with subsequent NAD in the market (linear regression with robust sandwich variance estimator, *t* = −0.00, *P* = 0.996, *R*^2^ < 0.001).

Cortisol at 14:00h was significantly correlated with our main measure of interest, *NAD* (*t* = 2.57, *P* = 0.037, *R*^*2*^ = 0.427), with *amplitude* (*t* = 2.84, *P* = 0.025, *R*^*2*^ = 0.356) and marginally with *RAD* (*t* = 2.17, *P* = 0.066, *R*^*2*^ = 0.316) in male and mixed markets but not in female-only markets (*P* > 0.3, see supplementary table 4). A simple linear prediction of pre-auction cortisol on *NAD* explains around 1/3 of the variability in male and mixed markets (*R*^*2*^ = 0.338, see Fig. 2). There was no correlation between 16:00h cortisol or testosterone levels and NAD, amplitude or RAD in any of the markets (*P* > 0.3).

Together, the associations found in this experiment support the hypothesis that cortisol is related to trading behaviour in the direction of greater risk-taking and mispricing at the market level. Of course, it is difficult to extrapolate experimental evidence to real world financial markets, and there have been mixed results in the literature regarding the ability of market prices to converge towards fundamental values^44^,^45^,^46^,^47^. Nevertheless, the fact that *ex-ante* cortisol was predictive of subsequent price instability is consistent with the hypothesis that variations in this hormone can have a destabilizing effect on financial markets^27^.

This evidence cannot be considered causal, since it relies solely on associations between differences in endogenous hormone levels and market behaviour. Moreover, it is difficult to interpret whether these associations reflect an effect of *elevated* cortisol (perhaps due to anticipatory stress) or of high baseline levels. It is also not clear whether pre-auction testosterone levels were close to baseline or elevated in anticipation to the experiment. We therefore cannot exclude the possibility that testosterone has significant effects on trading behaviour when it becomes elevated.

In order to test whether experimentally induced changes in these hormones directly affect financial trading behaviour we next investigated the effects of administering either cortisol or testosterone.

### Effects of administered cortisol or testosterone on male preferences for high volatility assets in a simulated trading environment

In two separate studies, healthy young male volunteers took part in a computerized trading simulator after being administered either cortisol or (in a different group) testosterone using a double-blind placebo-controlled balanced crossover design. Each subject was tested twice, once following hormone treatment and once following placebo. In the cortisol experiment, subjects were given a single dose of 100 mg cortisol orally (or placebo) and tested one hour later. In the testosterone experiment, they were administered three treatments of 10 g percutaneous 1% testosterone gel (or placebo) over a 48 hour period, and tested one hour after the last application. Both treatments induced significant increases in salivary levels of the respective steroid at the time of testing (see Fig. 1c,d and Methods), comparable with those previously reported in earlier administration studies^48^,^49^,^50^.

All tasks were conducted using real monetary incentives. Our aim was to measure risk-taking in a simplified context resembling the environment faced by professional traders in the stock market. Since the focus here was on individual rather than group behaviour, we used a trading simulator that borrowed features of earlier experimental designs to allow for greater experimenter control^51^,^52^. Subjects were shown plots of the price sequence for two ‘stocks’ and had to decide how much to invest in each over a total of 80 trials. The prices of both stocks were updated simultaneously at the end of every trial. During a trial, a subject had to [1] choose a stock, [2] enter an investment amount for that stock, [3] enter an investment amount for the alternate stock, [4] enter a guess about next period’s price for the first, and [5] for the second stock. Each decision had to be made within a 5 second time window.

**Figure 3:**
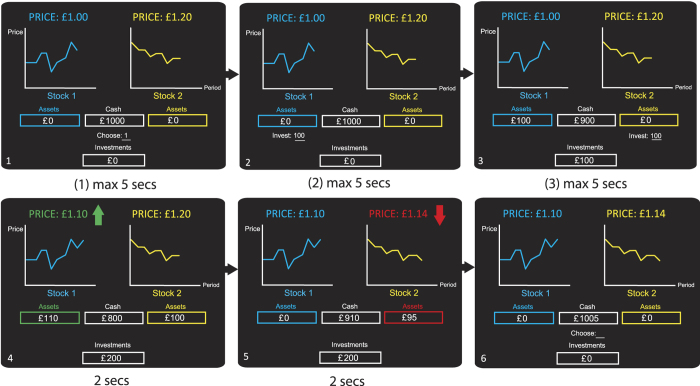
Schematic representation of the trading task (hormone administration experiments). After completing steps (1) to (3) and entering a guess for next period’s price (omitted in the figure), stock prices and cash balance are updated as shown.

Subjects began with an endowment of 10 GBP, and after every trial their endowment was updated according to their investment decisions and the new stock prices. Positive investments were profitable if the price of the stock went up but yielded losses when the price went down, with the reverse for negative investments (where the subject sells a borrowed asset that needs to be returned in the next period, i.e. a short-sale). The prices of both stocks followed two independent geometric random walks with positive drift. In any given trial, a stock could be in a high or low variance-return state (the drift and noise parameters of the random walks were high or low), with the state of each stock changing every 10 trials. As in a real financial market, subjects were not informed about the price generating process or the change of state; the only information on the price process available to them was a graph plotting the evolution of prices in real time (see Fig. 3 and supplementary material). As a measure of confidence before trading, participants were asked to guess their ranking within the group in terms of final trading profits.

### Effects of cortisol and testosterone on investment strategy

**Figure 4:**
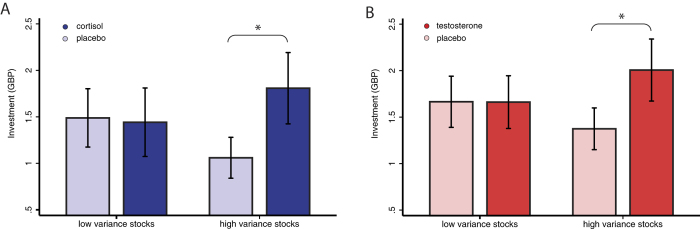
Mean net investment in low variance and high variance stocks (**A**) Cortisol administration experiment. Investment in high variance stocks was significantly increased following cortisol administration (*P* = 0.030) (**B**) Testosterone administration experiment. Investment in high variance stocks was also significantly increased following testosterone administration (*P* *=* 0.046).

We first examined whether either hormone was associated with changes in overall investments during the task. This revealed no effect of cortisol (Wilcoxon signed-ranks test, z = 0.72, *P* = 0.5) or testosterone (z = 1.16, *P* = 0.2) on overall mean investment amounts. Following our hypothesis, we then tested whether administered hormones specifically affected investments in high variance (riskier) stocks. We found that cortisol was associated with significantly increased mean investments in high variance stocks compared to placebo treatment (*z* = 2.17, *P* = 0.030), with investment in high variance stocks on average 70% higher in the cortisol condition (see Fig. 4a). We also found a significant increase of mean investments in high variance stocks following testosterone treatment compared to placebo (*z* = 2.00, *P = *0.046), with investment in high variance stocks on average 46% higher in the testosterone condition (see Fig. 4b). On the other hand, mean investments in low variance stocks did not significantly change following cortisol treatment (*z* = 0.595, *P* = 0.6) or testosterone treatment (*z* = 0.173, *P* = 0.9).

We next evaluated the robustness of these results to two important factors: first, learning could cause significant changes in behaviour from one week to the next; second, the length of the washout period of the administered hormone may have been insufficient to fully restore hormones levels or any associated behavioural effects back to baseline (see Methods). Therefore, we conducted a difference-in-differences analysis^53^ using a Mann-Whitney *U* test. This test compares the difference in investment from week 1 to week 2 between the ‘placebo-then-treatment’ group and the ‘treatment-then-placebo’ group. If the overall effect of treatment is to increase investment in high variance stocks, then the placebo-then-treatment group would exhibit a greater increase in investment from week 1 to week 2 than the treatment-then-placebo group, regardless of possible learning or carryover effects from treatment to placebo (see Methods for further details).

This test confirmed a significant positive effect of treatment on investment in high variance stocks, both for the cortisol administration study (*z* = 2.37, *P* = 0.018) and the testosterone administration study (*z* = 2.04, *P* = 0.041, see Fig. S2). Investments in high variance stocks were on average 13% lower in week 2 for the cortisol-then-placebo group. In contrast, the placebo-then-cortisol group increased their investments in week 2 by 177%. For the testosterone-then-placebo group, investments in high variance stocks fell on average 20% in week 2, whereas they increased by 88% on average in the placebo-then-testosterone group.

As the evidence from the market experiment indicated a significant association between *ex-ante* endogenous cortisol and investment behaviour, we examined whether pre-administration (endogenous) hormone levels moderated the effect of hormone treatment on investment (see supplementary table 5). Using fixed-effects panel regressions with mean investment as dependent variable, we found a significant interaction between pre-administration salivary cortisol levels and cortisol treatment (*P* < 0.05). Controlling for such interaction, cortisol administration yielded a significant effect on investment in high variance stocks (*t* = 3.32, *P* = 0.003). However, we found no significant evidence of an interaction between testosterone treatment and pre-administration salivary testosterone (*P* > 0.6). Previous studies have reported that second-to-fourth digit ratios (2D4D), a postulated indicator of prenatal exposure to testosterone^28^, predict success among high frequency traders^29^ and the likelihood of choosing a career in finance^54^. We tested whether this index might moderate the effect of testosterone administration on investment, but found no evidence of a significant interaction (*P* > 0.4).

### Possible pathways of the effect of cortisol and testosterone on investment behaviour

It is possible that the effect of cortisol or testosterone on investment behaviour was mediated by changes in price expectations, confidence, ability or attitude to risk.

We first assessed whether administration of either hormone was associated with a change in price expectations. We found no significant effect of cortisol on mean price expectations (Wilcoxon signed-ranks test z = 0.96, *P* = 0.3), but in contrast testosterone did have a significant effect on price expectations, with subjects predicting significantly larger price increases (z = 2.58, *P* = 0.01). In a more detailed analysis, we modelled price expectations as a function of previously observed price shocks and continued to find a significant effect of testosterone (see supplementary table 6).

We then used fixed effects panel regressions to analyse the effect of hormone administration on investment whilst controlling for expectations. These continued to show a significant effect of cortisol on investment in high variance stocks (*t* = 2.68, *P* = 0.012), indicating that price expectations do not explain the effect of cortisol on investment. However, the effect of testosterone no longer reached significance (*P* > 0.1), suggesting that testosterone may have affected investment behaviour by modulating optimism about future price changes (see supplementary table 7).

Administration of either hormone had no significant effect on subjects’ confidence in their trading ability relative to the other participants (Wilcoxon signed-ranks tests, cortisol vs placebo: *z* = 1.29, *P* = 0.2; testosterone vs placebo: *z* = 0.78, *P* = 0.4) or on their actual trading profits (cortisol vs placebo: *z* = 1.01, *P* = 0.3; testosterone vs placebo: *z* = 1.40, *P* > 0.1).

In sum, the increase in investments in high variance stocks caused by cortisol administration was not moderated by changes in subjects’ price expectations, confidence or ability. This suggests that cortisol directly affected subjects’ willingness to take risks. Testosterone, on the other hand, was associated with significantly increased optimism regarding price change expectations, making subjects more likely to expect stock prices to increase. The effect of testosterone on investment was no longer significant after controlling for price expectations, which suggests that increased optimism could be the mechanism through which testosterone affected investment behaviour.

## Discussion

Research in the behavioural sciences has long highlighted the important influence of hormonal variations in a wide variety of behaviours, yet their role in economic decision making has only begun to be examined.

Recent field evidence showed that endogenous cortisol was closely associated with market uncertainty and that testosterone was correlated with daily trading profits of professional high frequency traders^27^. It is therefore plausible that these two hormones exert an important influence on professional traders, who operate under highly competitive and stressful conditions. Our study aimed to test whether the hormonal variations observed in the field could, in turn, affect traders’ investment behaviour.

We showed that in men, elevated cortisol was associated with higher risk taking in experimental environments that resemble key aspects of real-world trading floors. In the first experiment, endogenous levels of cortisol were significantly associated with trading activity, mispricing and overall price instability in real multi-player asset markets. The causality of this association was established by the findings of the second experiment where investment in riskier stocks increased after cortisol administration. This effect was specific for high variance (riskier) stocks and remained significant after controlling for learning and price expectations, suggesting that the effect of cortisol did not operate merely through learning, general willingness to trade or beliefs, but rather, by increasing willingness to take risks. The fact that investment amounts increased specifically in the riskier stocks but not in low variance stocks may indicate that cortisol was particularly involved in affecting the decision of where to place the investment, rather than in how much to invest overall. We also found a significant interaction between treatment and baseline cortisol, suggesting that the treatment effect was particularly strong for subjects with elevated baseline levels of the hormone, again confirming the findings of the first experiment.

A strong association between cortisol levels and price volatility as indicated by bond futures has previously been reported in financial traders^27^. Here we show that traders with exogenously induced short-term elevations in cortisol adopt riskier investment strategies and that higher overall cortisol in the market predicts higher aggregate mispricing and volatility. Cortisol is a highly labile hormone where levels are rapidly altered in response to a variety of environmental stimuli, particularly demands that are perceived as threatening or uncontrollable^15^. Such properties make cortisol particularly suited for a role in modulating risk taking behaviour in response to external conditions. When professional traders undergo situations of high stress and elevated cortisol, such as before and after the release of important economic indicators^27^, raised cortisol might therefore encourage riskier trading. If riskier trading in turn destabilizes prices further, cortisol may exacerbate the stock market’s reaction to new information.

In the market experiment, cortisol levels were significantly higher at the start compared to the end of the session. This pattern is compatible with cortisol’s marked circadian rhythm^41^, but it may also reflect an effect of anticipatory stress caused by the prospect of participation in the experiment. It is therefore possible that the association between endogenous cortisol and trading behaviour reflected an effect of *elevated* levels of this hormone on behaviour.

The association between cortisol and risky trading behaviour in the market experiment was not present in women. This result is consistent with previous evidence of gender differences in the relationship between risk taking, cortisol and acute stress^30^,^55^. A recent study also reported that chronic elevations in baseline cortisol were associated with decreased risk taking and with more pronounced distortions of men’s weighting of probabilities relative to women^56^.

When considering previous cortisol administration studies more generally it is worth noting that more persistent elevations of cortisol and the associated loss of the normal daily cortisol rhythm may explain the different effects observed following chronic compared to acute treatments. Indeed, recent research has indicated that the effect of cortisol on behaviour varies over time, with both rapid and delayed effects^57^,^58^. The behavioural effects we observed in the cortisol administration experiment here are more likely due to the influence of acute effects of cortisol that have been linked with reduced attention to threats or fearful stimuli in healthy young men^59^,^60^, rather than the effects of chronically elevated cortisol which have been associated with increased risk aversion^55^,^60^.

Although the cortisol dose we employed is in the upper range of treatments used in the literature^31^,^48^,^61^, the findings reported here are in line with a previous cortisol administration study in which participants exhibited an increased preference for risk in a lottery task^31^. Related work has demonstrated that induced stress can also influence decision making^62^. Dependent on the specific task employed, induced stress can confer adaptive^55^, or maladaptive adjustments in behaviour^30^. However, it is important not to assume that all the effects of stress are related to cortisol, due to the wide variety of alterations it can cause in both physiology and neural activity, depending on the type of stress, its context, and the individual concerned^63^.

When we examined the relationship between testosterone and behaviour using the same tasks we found a slightly different picture. Previous studies have reported associations between circulatory testosterone levels and financial risk preferences^32^,^64^. However, we found no significant evidence associating endogenous testosterone levels with trading behaviour in multi-person markets. It is likely that behaviour in this environment is contingent on a greater number of factors than in the simpler tasks previously used to investigate the connection between testosterone and risk taking. This, in combination with the limitation of our sample size may account for our null finding. However, when we experimentally induced testosterone increases through direct administration, we did observe a significant effect on financial risk taking. Subjects invested larger amounts on the riskier stock after testosterone administration than after placebo. This effect operated partly through a change in price expectations, with testosterone inducing significantly more optimistic expectations about future price increases. These findings are consistent with recent evidence that endogenous *changes* in testosterone are predictive of subsequent risk taking behaviour^33^.

Testosterone is known to be responsive to a broad range of environmental stimuli, particularly those involving competition^16^,^23^,^26^. The associations between daily testosterone and profit levels observed in a field study of high frequency traders^27^ highlights that the possibility of an effect of this steroid hormone on financial decision making could be of great economic interest. For instance, the fact that winning or losing induces changes in the testosterone levels of fans at sporting events^20^ might help to explain why stock market returns respond to results of major sporting competitions^65^. More importantly, winning money in a competition or by chance has been shown to increase testosterone levels^16^,^17^,^33^. Our evidence shows that increases in testosterone lead to greater optimism and risk taking. In this way, testosterone may help to sustain the upward momentum of a bull market, in which high profits fuel optimism about future price increases and lead to further risk taking. Depending on the situation, this feedback mechanism could be maladaptive and encourage traders to “ride” a stock market bubble for too long.

We explored other factors that might moderate the behavioural response to testosterone. It has been suggested recently that there may be an interaction between the relative levels of endogenous testosterone and cortisol – the dual hormone hypothesis - that could influence risk taking behaviour^40^. We tested for such an effect in the market experiment but found no significant interaction in this instance. We also tested whether individual differences in the 2D4D ratio moderated the response to testosterone administration but found no evidence of a significant interaction. A factor that we were unable to examine was the contribution that genetic differences may have had on responsiveness to testosterone. The length of a polymorphic CAG repeat sequence in the androgen receptor gene is known to be inversely related to the transcriptional activity of the androgen receptor^66^. Therefore, it is possible that individual variability in genetic sensitivity to testosterone may have a significant influence on the behavioural response, and may have more explanatory power when measuring baseline levels of testosterone.

In this study we chose to only test men in the administration experiments since the vast majority of traders are male, and our aim was to relate our findings as closely as possible to the conditions of a financial trading environment. However, this decision presented a challenge since there are only a small number of testosterone administration studies in males in the literature to draw comparisons with^67^,^68^,^69^.

A possible limitation of the testosterone administration procedure we chose, which was a compromise solution based on our interest in recreating the winner effect^18^,^19^ and persistent elevations in testosterone observed in professional traders^27^, is that it may not have induced peak behavioural effects at the time point we collected the outcome measures. Additionally, the 1-week washout period between treatment and placebo may not have been sufficient to fully restore testosterone levels back to baseline. This means that the effects of testosterone might have been even stronger had we allowed for a longer interval between treatment and placebo. Finally, as there is evidence of both long and short term effects of testosterone on behaviour^25^,^68^, and more specifically evidence to suggest that the winner effect may not necessarily require multiple victories to become manifest^71^,^72^, it may have been possible to elicit such effects using a shorter administration protocol.

Although our findings suggest a role for both cortisol and testosterone in the instability of financial markets, identifying a neurobiological mechanism from this data is more challenging, particularly since the neural correlates of market behaviour have only begun to be investigated^13^,^52^,^73^. In a neuroimaging study where the effect of cortisol was examined via inducing stress in the participants, decreased activity in medial prefrontal cortex was observed in response to the presence of rewarding stimuli, but activity within ventral striatum was not affected^74^. On the other hand, direct administration of cortisol has been associated with reduced activity in striatum and amygdala in response to rewarding stimuli^75^. It is possible that this reflects two routes by which risk-seeking behaviour is modulated via changes in cortisol; bottom-up changes in baseline cortisol levels and top-down adjustments induced by external stressors, although further research is required to validate this possibility.

The neurobiology of the brain response to testosterone is less well understood, but recent work has shown that administration of testosterone in women is associated with increases in the differential brain response to stimuli associated with rewards and appetitive goal attainment in ventral striatum during reward anticipation^76^, reduced coupling of orbitofrontal cortex with amygdala^70^ and increased amygdala responses to untrustworthy faces^77^. A study which examined the neural basis of the optimism bias, the tendency to make overly confident predictions about the future, reported that optimism was related specifically to enhanced activation of the amygdala and rostral anterior cingulate cortex^78^. Therefore, it is possible that testosterone influences risk taking behaviour by altering activity within these regions and positively biasing predictions about the likelihood of future events, an effect reminiscent of our expectation-based pathway of testosterone action.

In conclusion, our experiments suggest that short-term alterations in male cortisol and testosterone levels have significant effects on financial decision making. The observed effects are compatible with field observations in professional traders and suggest that these hormones may play a destabilizing role in financial markets. Overall, our work suggests that stability in financial markets might be improved by considering how social, environmental and procedural factors such as the release of important financial information may impact the hormone levels of traders participating in those markets, and therefore could be of benefit to policymakers intent on developing more efficient institutions.

## Methods

All methods were carried out in accordance with the approved guidelines. All experimental protocols were approved by the Cambridge University Human Biology Research Ethics Committee and the Norfolk National Research Ethics Committee. Written informed consent was obtained from all subjects.

## Experimental asset market study

### Subjects

A total of 142 healthy men and women aged 18–30 participated in this study (69 men, 73 women, mean age = 21.9 yrs, SD = 2.85).

### Experimental procedure

Sessions were conducted with groups of typically 10 subjects in an open-plan computer lab (mean group size = 9.5, SD = 1.13). There were 4 male-only, 4 female-only and 7 mixed gender sessions in total. Subjects were allocated to computers separated by panels to prevent them from seeing the screens of other participants. They were asked not to communicate with other players during the experiment.

To minimize diurnal variation in hormones, all experimental sessions were conducted at 14:00. A total of three saliva samples were collected from each participant: one at the start of the session (14:00), one after the trading task (15:30), and one at the end of the session (16:00).

### The trading task

Subjects received paper instructions for the trading task (see “Instructions: asset market experiment” in the supplementary materials) and were asked to complete a 6-item questionnaire to test their understanding of the instructions. The trading task was programmed using z-tree^79^.

Markets were organized using a computerized double auction mechanism^35^,^36^,^37^. During a trading period, participants could submit any number of bids and asks, provided they had sufficient funds to complete the transaction. A bid (ask) consisted of an offer to buy (sell) a single asset at a specified price. For simplicity, we did not allow block trading (i.e. submitting bids or asks for multiple assets at a time). The market operated with an “open book”: all bids and asks submitted during a trading period were listed on subjects’ screens, anonymously and ordered by price. A subject could accept any number of bids or asks provided he or she had sufficient funds to complete the transaction.

Each trading period lasted 2 minutes. During trading, subjects could see all outstanding bids and asks in the market, all concluded transaction prices for that period, their current cash and asset holdings, and a plot of average transaction prices in every past period. At the end of a trading period dividends for that period were announced. These were the same for every asset in the market. Subjects were also provided with a summary of their total cash, assets and dividends up to that period. Before the new trading period began, subjects were asked to make a guess about the average transaction price in the next period. Each accurate guess was rewarded with an extra 10 pence at the end of the session. There were 15 trading periods in total, plus an additional practice period at the beginning.

Assets paid −24, −16, 4 or 36 units, called “francs” with equal probability at the end of every period, plus a maturity value of 360 francs at the end of period 15. Since dividends every period had zero expected value, the fundamental value of the asset was constant at 360 francs. This was clearly explained in the instructions, so that the fundamental value of the asset was known to all participants. At the start of the trading task, each subject received 10 assets and a 10,000 francs loan. Payoffs at the end of the trading task (in British pounds) were given by 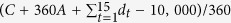, where *C* is final cash balance, *A* is final asset holdings, and *d*_*t*_ is total dividends or costs at period *t.* In order to maintain total available cash in the market constant, dividends were not added to subjects’ payoffs until the end. Francs had a conversion value of 360 francs = 1 GBP.

### Statistical analysis

To analyse associations between endogenous levels of cortisol or testosterone and trading behaviour in the experimental asset markets we estimated linear regressions separately for male and female traders where each subject provided a single observation of the dependent variable (mispricing or trading activity). To control for between-market variability, these regressions included dummy variables for each market and used a robust estimator of standard errors clustering by market. Finally, to check whether aggregate differences in endogenous cortisol or testosterone before each market were predictive of overall price instability, we estimated linear regressions with robust standard errors where each market provided a single observation of the dependent variable (either *NAD, RAD* or *amplitude*). For information on hormone analysis, refer to *salivary hormone analysis* below.

## Cortisol and testosterone administration studies

### Subjects

A total of 34 healthy men aged 18–30 were recruited to take part in the cortisol study. Four did not return for the second week of testing resulting in a total usable sample size of 30 participants. One additional outlier who earned 8–9 times more than other participants in the second session of the trading task was excluded from the analysis (this participant later admitted to being an experienced gambler) resulting in a sample size of 29 subjects (mean age = 25.7 yrs, SD = 2.68).

41 healthy men aged 18–30 were recruited for the testosterone study, four of whom did not complete both testing sessions. We excluded one additional outlier who in the second session invested >5 SD above the mean of our participant sample in high variance stocks and nearly doubled the second largest investor. This resulted in a total usable sample size of 36 (mean age 22.3, SD = 2.86). Participants were recruited on campus at the University of Cambridge via volunteer lists and online advertisements.

### Medical exclusion criteria

To minimize the risks of possible interactions with the administration of either hormone, a qualified clinician carried out all screening procedures, recording standard measures (blood pressure, height, and weight) and remained available throughout the experiment for medical support. Exclusion criteria were a personal history of heart disease, high blood pressure, diabetes, breathing problems (including asthma), skin sensitivities (including eczema), endocrine or hormone disorders, eye disease (including glaucoma), prostate disorders, liver or kidney impairment, neurological or psychiatric problems (including alcoholism, depression, schizophrenia or bipolar disorder), epilepsy, family history of heart arrhythmia or sudden death syndrome, head injury, recent major surgery, smoking or recreational drug use. In the cortisol study participants were also screened using the Beck’s Depression Inventory (BDI) and the profile of mood questionnaire (PoM) for symptoms of depression. No participant exceeded rejection threshold scores on either test (14+ for BDI and 30+ for PoM).

### Experimental procedure

The experiment employed a within-subjects, double-blind placebo-controlled balanced crossover design. Testing was divided into two sessions that took place at least one week apart, each lasting approximately 3 hours. In order to minimize differences in endogenous hormone levels due to diurnal variation, both sessions were conducted at the same time of the day for each participant. Due to unforeseen circumstances one participant in the testosterone study was tested in the morning of the first week and in the afternoon in the second session. The experiments were conducted at the Herchel Smith Building for Brain and Mind Sciences at the University of Cambridge.

Subjects were instructed not to eat or drink 30 minutes before each session. Once they arrived for testing and all screening checks had been passed they were asked to provide a baseline saliva sample.

### Drug Administration

In the cortisol study, following the baseline saliva sample, participants were administered a single tablet containing 100 mg of hydrocortisone or a placebo. Behavioural testing began 1 hour after administration. This approach has previously been employed to elicit consistent increases in cortisol over this time period^48^. Three further saliva samples were then collected at hourly intervals.

In the testosterone study, participants were administered either 10 g of Testogel^TM^ (1% testosterone gel) or a placebo of colourless hydroalcoholic gel which was applied to the shoulders. We chose a transdermal application method rather than via injection as this approach is less invasive and can be self-administered by the subjects at home. Although the time course of the effects of testosterone on behaviour in men are currently under research, pharmacokinetics of transdermal application have been investigated and are known to elevate testosterone levels for at least 12 hours following administration^49^,^50^,^80^. Furthermore, recent studies have reported significant changes in behaviour following testosterone loading periods of around 24 hours^67^,^69^. Each subject received a total of three testosterone or placebo doses prior to each experimental session: the first 48 hours before testing, the second 24 hours before testing (which the subject was given to apply at home), and the third one hour before the testing session. The participants confirmed that the gel they administered at home was applied at the same time of day as in the first session, and were made aware that they would be asked about this at the behavioural session. Additional saliva samples were collected when participants returned for the experimental session prior to the third administered dose and after participating in the trading task.

The choice of treatment regimens was motivated by the findings of Coates & Herbert (2008) which showed that traders exhibited extended periods where testosterone levels were raised on consecutive days, in line with increases in profits. This association appears to mirror the winner effect, also observed in the animal literature, whereby victories in competition for mates or food have been associated with elevated testosterone levels, and increased aggression^18^,^19^.

Based on these data, our hypothesis was that the effects of testosterone on economic decision making would become more prominent if endogenous levels were elevated over a longer period of time, rather than a short-term elevation. Although the timescale of the behavioural effects of testosterone has been well described in women^81^,^82^,^83^, in males there is currently comparatively little data on when the behavioural effects are maximal, particularly with respect to risk taking^67^,^68^,^69^. Therefore we used an administration procedure which would result in significant elevations of testosterone over a 48 hour period prior to testing in order to mimic the sustained elevation in testosterone associated with the winner effect and reported during traders’ winning streaks^27^.

The motivation for the cortisol administration scheme was different, as we wished to recreate the acute stress-related changes in cortisol that occur following market shocks or the release of important economic indicators such as US employment statistics. Although the dose is at the upper end of the range of doses used in the literature^31^,^48^,^61^, our aim was to model the behavioural response to major shocks in financial markets. The individuals who trade in these markets personally bear enormous financial responsibilities, such that large shocks in these markets can place them in extremely stressful situations. Our aim was to employ a dose at the upper end of the doses used in the literature in order to reflect the hormonal conditions likely to be present in trading floors during such events.

The timing of the behavioural measures with respect to the administrations reflects the best compromise solution we were able to achieve given the currently available information about the behavioural effects of administering testosterone in men^67^,^68^,^69^. We decided to standardise the experience of the participants across both experiments.

As behavioural effects have previously been reported 1 hour after cortisol administration^31^, we decided to match that profile following the final dose in the testosterone administration study.

Neither experimenter nor participants were aware of the order of administration at the time of testing in either experiment, which was previously randomized by the pharmaceuticals provider (Cardiff and Vale NHS Pharmacy, UK). The participants did not report any side-effects following administration of either drug or placebo in either experiment, and did not perform significantly better than chance when asked to guess in which session they received the active substance for the cortisol (*P* > 0.46) or testosterone (*P* > 0.62) experiments (two-sided binomial test).

### Salivary hormone analysis

Saliva specimens of 3 ml were collected by passive drool using 12 ml plastic reagent tubes (Sarstedt, UK) and immediately frozen at −80°C. Samples were analysed at the Salimetrics Centre of Excellence saliva laboratory in Cambridge (Salimetrics Europe) using a competitive immunoassay. Each assay was performed in duplicate, with inter- and intra-assay variations < 6%. Of the total number of samples collected (1,296), ~7% were excluded or could not be analysed due to either insufficient saliva volume, likely interference with the assay or exceeding the upper limit of sensitivity for the assay. Evidence from a recent study which employed a similar testosterone administration procedure and reported saliva concentrations above 1000 pg/ml^50^ demonstrates that this administration approach induces levels of circulating unbound testosterone which can exceed the range of standard assays.

For statistical purposes, individual salivary hormone data is log-transformed in order to adjust for the non-normality of the data. The data is then analysed using two-way repeated measures ANOVA. In the cortisol study, we found a significant time effect (*F* = 50.80; *P* < .0001), drug effect (*F* = 292.25; *P* < .0001) and drug-time interaction effect (*F* = 190.09; *P* < .0001). Further paired t-tests show a significant time effect under placebo only in the first hour (*P* < .0001) but not in subsequent sampling times (*P* > .2); a significant time effect in every hour under treatment (*P* < .01); and a significant treatment effect in every period following administration (*P* < .0001) but not before administration (*P* = .5). In the testosterone study, we found a significant time effect (F = 55.22; *P* < .0001), drug effect (*F* = 50.99; *P* < .0001) and drug-time interaction effect (*F* = 32.70; *P* < .0001). Further paired t-tests show no significant time effect under placebo (first 48 hours: *P* = .4; last 2 hours: *P* = .08); a significant time effect under treatment (first 48 hours: p < .0001; last 2 hours: *P* = .1); and a significant treatment effect 48 hr and 50 hr after administration (48 hr: *P* < .0001; 50 hr: *P* = .0001). We also found a significant treatment effect at 0 hr (*P* < .001), suggesting that the washout period of one week was insufficient to fully restore testosterone levels back to baseline. Indeed, under placebo, subjects in the testosterone-placebo condition had significantly higher 0 h testosterone than subjects in the placebo-testosterone condition (*P* = .02) whereas there was no significant difference between both groups at 0 h under testosterone (*P* = .14). To address this issue, we performed additional statistical tests detailed in *Statistical Analysis* below. In the market study, we performed repeated measures ANOVA tests with gender as between-subjects factor. For cortisol, we found a significant time effect (*F* = 90.40; *P* < .0001), no gender effect (*F* = .14; *P* = . 7) or gender-time interaction effect (*F* = .11; *P* = .7). Further paired t-tests show significant time effects at both intervals (*P* < .01). For testosterone, we found a significant time effect (*F* = 10.37; *P* < .01), gender effect (*F* = 108.38; *P* < .0001), and no significant gender-time interaction effect (*F* = .22; *P* = .6). Further t-tests show a significant time effect from 14:00 to 15:30 (*P* < .01) and a significant gender effect at all times (*P* < .0001).

### 2D4D digit ratio measurement

2D4D ratios were measured using a previously published methodology^29^. Briefly, to determine 2D4D we used a high resolution flatbed scanner to generate an image of the participants’ right-hand and measured digit length from the metacarpophalangeal crease to the finger tip. A handprint was acquired during both behavioural visits to provide an average measurement. The handprints for both sessions were measured digitally for 2D4D and the mean value taken.

### Trading task

Subjects received paper instructions for the trading task and were asked to complete a 3-item questionnaire to test their understanding of the instructions. If they gave an incorrect answer, an explanation was provided and the question was asked again. In order to provide an estimate of confidence before trading, participants were asked to guess where they expected to rank within the group in terms of final trading profits. The task was programmed using Presentation software (Neurobehavioral Systems, Inc.) and presented on a 17inch CRT monitor.

In the trading task subjects were shown price plots of two “stocks” and had to decide how much to invest in each of them over a total of 80 trials. The price of both stocks was updated simultaneously at the end of every trial. During a trial, the subject: [1] chose a stock, [2] entered an investment amount for that stock, [3] entered an investment amount for the other stock, [4] entered a guess about next period’s price for the first stock, and [5] entered a guess about next period’s price for the other stock. The participants were given a maximum of 5 seconds to make each decision, but could respond faster if they chose to. Hence, each trial could last at most 25 seconds.

A subject could invest any positive amount on either stock up to their current cash endowment; they could also “short-sell” any negative amount up to their current cash endowment. By short-selling, a subject received cash in advance for the sale of stocks they did not yet own and which had to be bought in the following period. Lastly, they could also invest zero. These actions could all be executed by entering a positive number, a negative number (both in steps of [2] or [3]), or “0”. At the end of each trial, the purchases or sales made by the subject were cleared so that the subject’s portfolio returned to zero stock holdings. The cash endowment in the next period depended on whether prices in each stock rose or fell and on whether the subject had invested or short-sold either stock. The profits or losses resulting from an investment (or short-sale) on the *i*^th^ stock were given by:


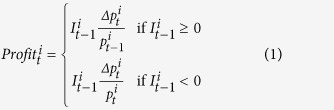


where *i* is stock number, *i*∈{1,2}, t is the trial number, *t*∈{1,…,80}, *I*^*i*^ is amount invested in stock *i*, with *I*^*i*^ > 0 indicating purchases and *I*^*i*^ < 0 indicating short-sales. Hence, the total change in cash from one trial to the next was given by:


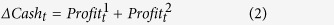


A positive investment in trial *t*−1 consisted of purchasing an amount of assets at price *p*_*t*−1_ and reselling these assets in trial *t* at price *p*_*t*_. A negative investment (in this case a short-sale) in trial *t*−1 consisted of committing to sell assets in trial *t* at price *p*_*t*−1_. In the case of short-selling, the subject received the money from the short sale in trial *t*−1 and had to buy the assets at price *p*_*t*_ at the end of trial *t* to restore his short position. Consequently, positive investments were profitable when the price of a stock increased and short-sales were profitable when its price dropped.

Subjects could not invest more than their current cash endowment; however, by short-selling, subjects could “borrow” additional cash. To minimize the risk of bankruptcy, we did not allow subjects to short-sell an amount larger than their cash holdings at the start of each trial. This meant that a subject could increase their cash available for investment on one of the stocks by at most 100% by first short-selling the other stock. Subjects started with an endowment of 1000 units of cash. The exchange rate was 100 units = 1 GBP.

The prices of both stocks followed two independent geometric random walks with drift. At any point in time, a stock could be in a *high return* or a *low return* state and in a *high variance* or a *low variance* state. Stocks stayed at a given return and variance state for 10 trials in a row. Each combination of states of both stocks occurred at least once, in random order. Subjects, however, were not informed about any aspect of the price generating process.

The most relevant combination of states, in terms of decision conflict, arose when one of the stocks was in the *low return, low variance* state and the other was in the *high return, high variance* state. For this reason, this combination of states occurred three times during a session, whereas every other combination of states occurred only once.

The specific equation used to generate the price process was:


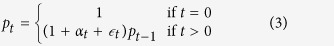


where *α* ∈ {0.003,0.011}, depending on whether the stock was in a low return state or a high return state, and *𝜖*_*t*_ ~ *U*[*a*, *b*] with (a= – 0.04; b = 0.04) in low variance states and (a= – 0.08; b = 0.08) in high variance states.

Substituting *p*_*t*_ in equation 3, we get:


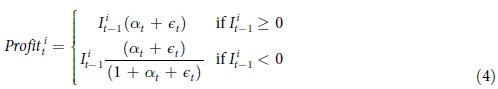


Hence, the expected marginal return from investment was *α*_*t*_, whereas the expected marginal return from short-selling was −*α*_*t*_/(1 + *α*_*t*_).

The money earned in this task was equal to the subject’s final cash balance at the end of trial 80. Subjects were also rewarded an additional sum proportional to their average price guessing accuracy. Price guesses were elicited at the end of every trial. Thus, at trial *t* subjects had to enter a guess about *p*_*t*+1_ for stock 1 and for stock 2.

### Presentation

A picture of the task as seen by the participant is displayed in the instructions (see “Instructions: trading simulator” in the supplementary materials). Prices of each stock were plotted with the current price always at a fixed y-coordinate in the graph, so that price changes were represented by shifts in the trail of past prices. This was in order to ensure that plots could never go above or below the bounds of the screen, while maintaining a fixed scale in the graph. After the 10^th^ trial, the current price remained at a fixed x-coordinate, so that from then on, only prices (*p*_*t*_,…,*p*_*t−9*_) were displayed.

### Statistical Analysis

Since the cortisol and testosterone administration studies used within-subjects designs, our primary analysis employed Wilcoxon signed-ranks test. This is a non-parametric paired difference test appropriate for relatively small samples and which does not require data to be normally distributed. To ensure that every paired observation was independent, we conducted this test using a single observation from each subject and treatment (e.g. we compared 36 observations of average investment under placebo with 36 observations of average investment under testosterone). In our secondary analysis we checked whether our primary findings held after controlling for additional factors such as learning and treatment order effects. This was done using a difference-in-differences analysis^53^. Using Mann-Whitney *U* tests, we tested whether the mean increase in investment from week 1 to week 2 was larger for subjects receiving placebo-then-treatment than for subjects receiving treatment-then-placebo. Let mean investments, *y*, be a linear function of treatment and experience, and allow for a possible additional washout effect for subjects receiving placebo in week 2, such that *E*[*y*|*t*,*e*,*w*] = *β*_0_ + *β*_1_*t* + *β*_2_*e* + *β*_3_*w*, where *t* = 1 if hormone, *t* = 0 if placebo; *e* = 1 if week 2, *e* = 0 if week 1; *w* = 1 if placebo in week 2, *w* = 0 otherwise. The difference between investments in week 2 and week 1 for the placebo-then-treatment group, Δ*E*[*y*](placebo-then-treatment) is then *β*_1_ + *β*_2_. Similarly, the difference for the treatment-then-placebo group, Δ*E*[*y*](treatment-then-placebo) is −*β*_1_ + *β*_2_ + *β*_3_. Taking the difference between these two expressions gives 2*β*_1_−*β*_3_. Our statistical hypothesis is that 2*β*_1_−*β*_3_ > 0. Given the reasonable assumption that the effect of washout can at most be as big as the effect of treatment, then this inequality will hold if and only if the effect of treatment, *β*_1_, is positive. To test for possible interactions with baseline hormone levels or digit ratios, and to analyse price expectations, we estimated fixed-effects panel regressions (within-subjects estimation) with robust standard errors (Huber/White/sandwich estimator) clustered by subject. This method is appropriate for analysing data with repeated observations and multiple time-varying explanatory variables. Standard errors were computed using a conservative estimator robust to serial correlation in the error terms. As an additional check, we examined any potential differences in behaviour between placebo groups in either study using Mann-Whitney *U* tests. We found no significant differences in overall investments (*P* = .4), investments in low variance stocks (*P* = .5), or investments in high variance stocks (*P* = .5).

## Additional Information

**How to cite this article**: Cueva, C. *et al.* Cortisol and testosterone increase financial risk taking and may destabilize markets. *Sci. Rep.* **5**, 11206; doi: 10.1038/srep11206 (2015).

## Supplementary Material

Supplementary Information
