# Supplementary material for: Cortisol and testosterone increase financial risk taking and may destabilize markets
Source: Sci Rep. 2015 Jul 2;5:11206. doi: 10.1038/srep11206 (PMC4489095; doi:10.1038/srep11206)
Supplement: Supplementary Information [file srep11206-s1.pdf]

Supporting Online Material

Cortisol and testosterone increase financial risk taking and may destabilize markets

Authors: Carlos Cueva[a,1], R Edward Roberts[b,1], Tom Spencer[c,d], Nisha Rani[d], Michelle Tempest[e], Philippe N Tobler[f], Joe Herbert[g], Aldo Rustichini[h,i,2]

Affiliations:

[a] Departamento de Fundamentos del Análisis Económico, Universidad de Alicante, Spain

[b] Division of Brain Sciences, Department of Medicine, Imperial College London, UK

[c] Department of Psychiatry, University of Cambridge, UK

[d] Cambridgeshire and Peterborough NHS Foundation Trust, Elizabeth House, Fulbourn Hospital, Cambridge, UK

[e] Royal College of Psychiatrists, UK

[f] Laboratory for Social and Neural Systems Research, Department of Economics, University of Zurich, Switzerland

[g] John van Geest Centre for Brain Repair, Department of Clinical Neurosciences, University of Cambridge, UK

[h] Department of Economics, University of Minnesota, USA

[i] Department of Physiology, Development and Neuroscience, University of Cambridge, UK

[†] C.C. and R.E.R contributed equally to this work.

[2] **Contact:** arust@econ.umn.edu

Tables and Figures

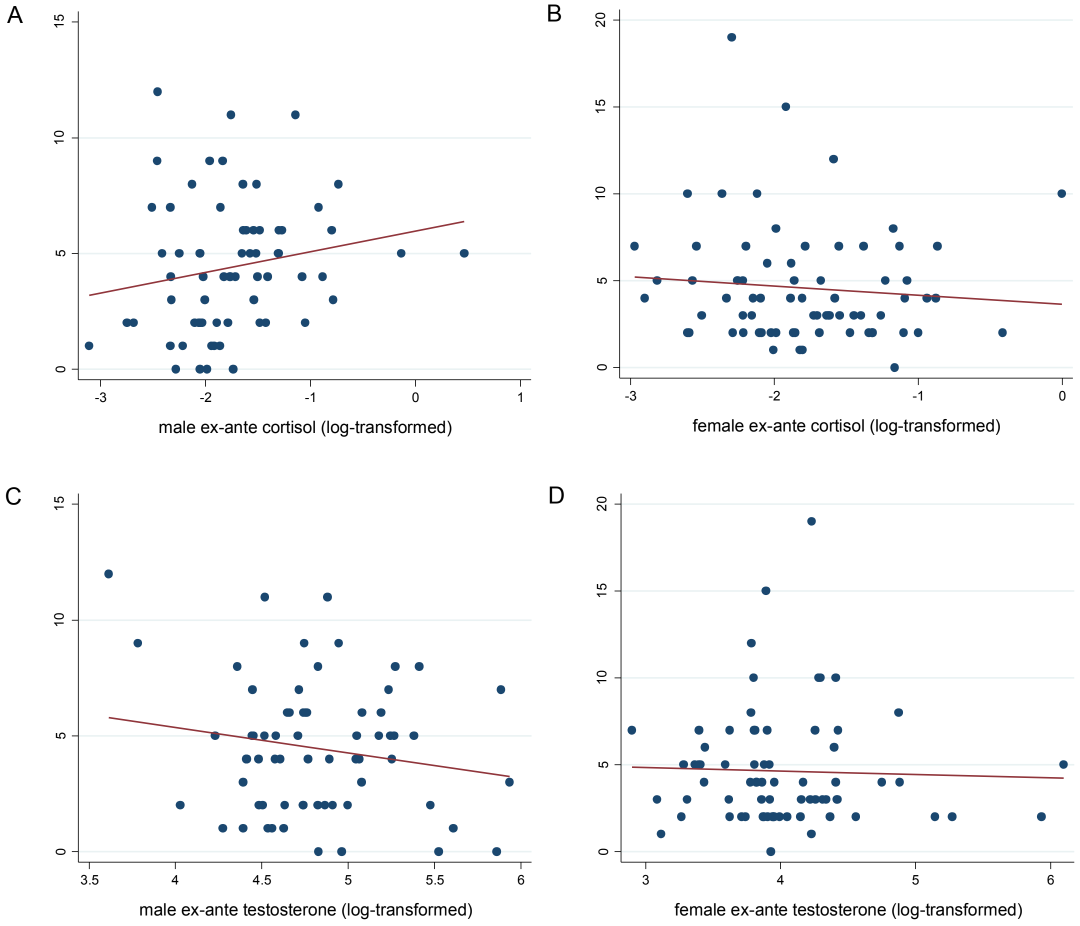

Figure S1. Ex-ante salivary hormone concentrations (log-transformed) and trading activity in the first period of Experiment 1. **(A)** Male trading activity and salivary cortisol (r = .897; p =.122; R^2 = 0.036; N = 68) **(B)** Female trading activity and salivary cortisol (r = -.528; p =.443; R^2 = 0.008; N = 72) **(C)** Male trading activity and salivary testosterone (r = -1.092; p =.162; R^2 = 0.030; N = 66) **(D)** Female trading activity and salivary testosterone (r = -.196; p =.778; R^2 = 0.001; N = 71).

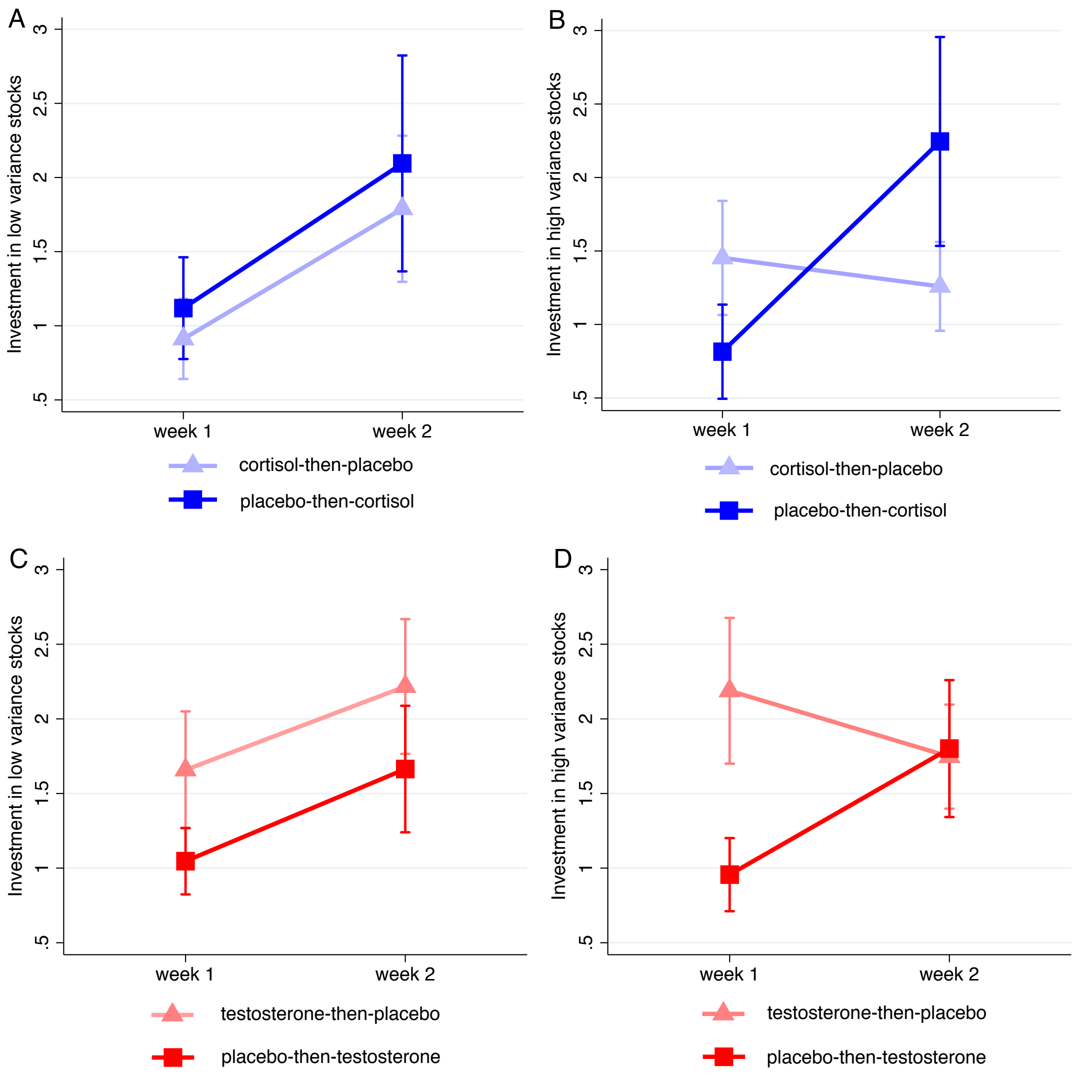

Figure S2. Average net investment in low and high variance stocks. **(A)** Cortisol administration experiment, low variance stocks – treatment order had no significant effect on the difference between week 1 and week 2 investments (Mann-Whitney U-test of difference in differences, z = 0.40, *P* = 0.7). **(B)** Cortisol administration experiment, high variance stocks - subjects receiving placebo-then-cortisol significantly increased their investments in week 2 compared to subjects receiving testosterone-then-placebo (z = 2.37, *P* = 0.018). **(C)** Testosterone administration experiment, low variance stocks – treatment order had no significant effect on the difference between week 1 and week 2 investments (z = 0.05, *P* > 0.9). **(D)** Testosterone administration experiment, high variance stocks – subjects receiving placebo-then-testosterone significantly increased their investments in week 2 compared to subjects receiving testosterone-then-placebo (z = 2.04, *P* = 0.041).

Table S1. Trading activity and mispricing in the first period. Linear regressions with robust standard errors (Huber/White/sandwich estimator, clustered by market). Pre-auction hormone data log-transformed.

(A) Individual trading activity (number of transactions).

(B) Individual mispricing $= \sum_{p_{buy}>1} (p_{buy} - 1) + \sum_{p_{sell}<1} (1 - p_{sell})$,

where p_{buy} and p_{sell} are buying and selling prices, respectively.

**A**	**men**		**women**		**pooled**
	b (se)	b (se)	b (se)	b (se)	b (se)
**cortisol**	-4.718	1.541**	-0.104	-1.275	-1.102
	(8.006)	(.354)	(2.947)	(.653)	(.744)
**testosterone**	0.695	-1.673	-0.254	0.299	0.163
	(2.538)	(.947)	(1.784)	(.638)	(.490)
**cortisol x testosterone**	1.256		-0.289		
	(1.637)		(.682)		
**male**					16.028*
					(5.650)
**cortisol x male**					2.709*
					(1.028)
**testosterone x male**					-2.388*
					(.825)
session dummies	.	.	.	.	.
(omitted)	(.)	(.)	(.)	(.)	(.)
**Num. of observations**	66	66	71	71	137

**B**	**men**		**women**		**pooled**
	b (se)	b (se)	b (se)	b (se)	b (se)
**cortisol**	-1.579	0.143	-0.344	-0.094	-0.069
	(1.223)	(.093)	(.400)	(.063)	(.055)
**testosterone**	0.485	-0.166	0.161	0.043	0.013
	(.417)	(.133)	(.084)	(.057)	(.049)
**cortisol x testosterone**	0.345		0.062		
	(.251)		(.084)		
**male**					1.306
					(.701)
**cortisol x male**					0.208
					(.104)
**testosterone x male**					-0.208
					(.119)
session dummies	.	.	.	.	.
(omitted)	(.)	(.)	(.)	(.)	(.)
**Num. of observations**	66	66	71	71	137

*p<0.05, **p<0.01

Table S2. Trading activity and mispricing of male and female traders throughout the asset market study. Random-effects GLS regressions with robust standard errors (Huber/White/sandwich estimator, clustered by market). **(A)** Individual trading activity. **(B)** Individual mispricing. Hormone data log-transformed.

**A**	**men**		**women**	
	b (se)	b (se)	b (se)	b (se)
**cortisol (14:00)**	-0.157		-1.438	
	(.476)		(.850)	
**testosterone (14:00)**	-0.446		-0.318	
	(.391)		(.466)	
**cortisol (16:00)**	1.073*	.909*	-0.400	-1.119
	(.452)	(.440)	(.850)	(1.047)
**testosterone (16:00)**	0.034	-0.006	0.036	0.234
	(.370)	(.469)	(.701)	(.528)
session dummies	.	.	.	.
(omitted)	(.)	(.)	(.)	(.)
**Num. of groups**	65	66	70	71
**Num. of observations**	975	990	980	995

**B**	**men**		**women**	
	b (se)	b (se)	b (se)	b (se)
**cortisol (14:00)**	-0.019		-0.077	
	(.018)		(.048)	
**testosterone (14:00)**	0.044		0.044	
	(.037)		(.045)	
**cortisol (16:00)**	0.042*	0.039*	-0.082	-0.116
	(.019)	(.017)	(.069)	(.080)
**testosterone (16:00)**	-0.047	-0.024	-0.028	0.025
	(.024)	(.025)	(.044)	(.034)
session dummies	.	.	.	.
(omitted)	(.)	(.)	(.)	(.)
**Num. of groups**	65	66	70	71
**Num. of observations**	975	990	980	995

Table S3. Final trading profits in the asset market study. Linear regressions with robust standard errors (Huber/White/sandwich estimator, clustered by market). Hormone data log-transformed.

	**men**		**women**	
	b (se)	b (se)	b (se)	b (se)
**cortisol (14:00)**	0.751	0.739	0.651	1.226
	(.866)	(.647)	(.414)	(.626)
**testosterone (14:00)**	-0.790	-1.077	-1.313	-0.721
	(.418)	(.808)	(1.287)	(.534)
**cortisol (16:00)**	-0.213		1.707	
	(.646)		(1.247)	
**testosterone (16:00)**	-0.457		0.623	
	(.601)		(.955)	
session dummies	.	.	.	.
(omitted)	(.)	(.)	(.)	(.)
**Num. of observations**	66	66	71	71

Table S4. Aggregate price instability in the market. Linear regressions with robust standard errors (Huber/White/sandwich estimator). Mixed = 1 if group is mixed-gender and = 0 if group is male-only. Average (normalized) hormone levels in the market. **(A)** Normalized absolute deviation, NAD. **(B)** Amplitude. **(C)** Relative absolute deviation, RAD.

**(A) NAD[1]**	b (se)	b (se)	b (se)	b (se)
**cortisol (14:00)**	1.414	1.447*	1.452*	1.417*
	(.734)	(.56)	(.520)	(.538)
**testosterone (14:00)**	-0.594	-0.628	-0.257	
	(.815)	(.571)	(.699)	
**cortisol (16:00)**	0.134			
	(2.161)			
**testosterone (16:00)**	0.134			
	(2.161)			
**mixed**	-1.516	-1.595		
	(2.446)	(1.564)		

**(B) Amplitude[2]**	b(se)	b(se)	b (se)	b (se)
**cortisol (14:00)**	0.006	0.014*	0.014	0.015
	(.012)	(.005)	(.007)	(.008)
**testosterone (14:00)**	0.047	0.000	0.007	
	(.065)	(.008)	(.013)	
**cortisol (16:00)**	-0.007			
	(.022)			
**testosterone (16:00)**	-0.049			
	(.058)			
**mixed**	-0.018	-0.027		
	(.027)	(.022)		

**(C) RAD[3]**	b (se)	b (se)	b (se)	b (se)
**cortisol (14:00)**	0.005	0.009	0.009	0.010
	(.011)	(0.004)	(0.008)	(0.007)
**testosterone (14:00)**	0.023	-0.006	0.001	
	(.063)	(0.007)	(0.011)	
**cortisol (16:00)**	-0.002			
	(.022)			
**testosterone (16:00)**	-0.030			
	(.057)			
**mixed**	-0.028	-0.032		
	(0.032)	(0.022)		
**Num. of observations**	11	11	11	11

* $p<0.05$. [1] $NAD = \sum_t \sum_i |p_{i,t} - 1|/A$, where $p_{i,t}$ is the price of the ith transaction in period t and A is a constant scaling factor. [2] $Amplitude = (\max(P_t) - \min(P_t)/F$, where P_t is the median transaction price at trading period t, and F is the fundamental value of the asset (1 GBP). [3] $RAD = {}^1/_N \sum_t |\ P_t - F|/F$, where P_t is the median transaction price at trading period t.

Table S5. Interactions between treatment and baseline hormone levels or second-to-fourth-digit ratios (2D4D). Fixed-effects panel regressions (within-subjects estimation) with robust standard errors (Huber/White/sandwich estimator, clustered by subject). The regressions labelled *highvar only* and *lowvar only* estimate the same model within subsets of the data corresponding to high variance stocks and low variance stocks respectively, excluding the first two observations after a state change. **(A)** Cortisol administration study. **(B)** Testosterone administration study.

**A**	**highvar only**	**lowvar only**
	b (se)	b (se)
**cortisol admin.**	338.3**	273.1
	(102.0)	(138.2)
**baseline cortisol**	-117.2	-188.7
	(84.52)	(96.84)
**admin x baseline**	150.1*	167.0*
**cortisol**	(53.78)	(79.38)
**session**	43.19	75.32*
	(24.41)	(33.15)
**Num. of groups**	24	24
**Num. of obs.**	48	48

**B**	**highvar only**		**lowvar only**	
	b (se)	b (se)	b (se)	b (se)
**testo admin.**	-415.9	-466.7	240.9	62.44
	(1,041)	(771.2)	(1,067)	(830.8)
**baseline testo**	16.78		-76.87	
	(55.44)		(43.61)	
**admin x baseline**	11.95		28.45	
**testo**	(65.28)		(73.67)	
**admin x 2D4D**	455.1	562.7	-465.0	-59.40
	(1,011)	(810.9)	(935.6)	(866.6)
**session**	21.75	24.63	96.63**	61.81*
	(41.93)	(27.11)	(32.68)	(28.41)
**Num. of groups**	35	35	35	35
**Num. of obs.**	64	70	64	70

Table S6. Price change expectations. Fixed-effects panel regressions (within-subjects estimation) with robust standard errors (Huber/White/sandwich estimator, clustered by subject). Price change is defined as $100 \times (price_t - price_{t-1}) / price_{t-1}$. Price change expectation is $100 \times (E_t(price_{t+1}) - price_t) / price_t$. High variance state = 1 if stock is in a high variance state, = 0 otherwise. First two trials after each state change excluded. **(A)** Cortisol administration study. **(B)** Testosterone administration study.

A

	b (se)	b (se)	b (se)	b (se)
**cortisol**	-0.175	-0.0545	-0.0545	-0.0516
	(0.221)	(0.239)	(0.239)	(0.239)
**price change**	0.229***	0.229***	0.229***	0.228***
	(0.0248)	(0.0248)	(0.0246)	(0.0249)
**trial**	-0.00660*	-0.00660*	-0.00660*	
	(0.00278)	(0.00278)	(0.00275)	
**high variance state**	-0.134	-0.0130		
	(0.145)	(0.109)		
**cortisol × high variance state**	0.239			
	(0.172)			
**Num. of groups**	29	29	29	29
**Num. of obs.**	6,841	6,841	6,841	6,841

* *p*<0.05, ** *p*<0.01, *** *p*<0.001

B

	b (se)	b (se)	b (se)	b (se)
**testosterone**	0.229	0.281*	0.282*	0.281*
	(0.148)	(0.117)	(0.117)	(0.117)
**price change**	0.261***	0.261***	0.260***	0.260***
	(0.0351)	(0.0352)	(0.0351)	(0.0351)
**trial**	-0.00289	-0.00287	-0.00270	
	(0.00193)	(0.00194)	(0.00193)	
**High variance state**	-0.204	-0.151		
	(0.145)	(0.104)		
**testosterone × high variance state**	0.106			
	(0.149)			
**Num. of groups**	36	36	36	36
**Num. of obs.**	8,489	8,489	8,489	8,489

* *p*<0.05, ** *p*<0.01, *** *p*<0.001

Table S7. Investment in each trial of experiment 2. Fixed-effects panel regressions (within-subjects estimation) with robust standard errors (Huber/White/sandwich estimator, clustered by subject). The first regression (pooled) is estimated from the full data set, excluding the first two observations after a state change. The second and third regressions (*highvar only* and *lowvar only*) estimate the same model within subsets of the data corresponding to high variance stocks and low variance stocks respectively. Guessed price change is defined as price change expectations in table S7. **(A)** Cortisol administration study. **(B)** Testosterone administration study.

**A**	**pooled**	**highvar only**	**lowvar only**
	b (se)	b (se)	b (se)
**cortisol**	59.89	93.58*	27.80
	(34.01)	(34.91)	(37.14)
**guessed price change**	71.73***	73.91***	66.91***
	(15.40)	(16.25)	(14.83)
**trial**	2.051**	2.124**	2.156**
	(0.564)	(0.682)	(0.623)
**session**	86.95*	74.97*	90.64*
	(34.05)	(35.47)	(36.64)
**Num. of observations**	8,446	3,433	3,408
**Num. of groups**	29	29	29

**B**	**pooled**	**highvar only**	**lowvar only**
	b (se)	b (se)	b (se)
**testosterone**	11.10	32.92	-9.812
	(16.88)	(20.86)	(25.34)
**guessed price change**	82.85***	83.91***	81.50***
	(11.48)	(12.02)	(12.43)
**trial**	1.666***	1.451***	2.008***
	(0.369)	(0.359)	(0.534)
**session**	50.46**	30.68	69.75**
	(17.12)	(22.08)	(25.14)
**Num. of observations**	8,489	4,234	4,255
**Num. of groups**	36	36	36

* p<0.05, ** p<0.01, *** p<0.001

Instructions: asset market experiment

This experiment aims to emulate a stock market environment in which you will be able to buy and sell assets. You are free to decide how to play in order to maximize your earnings.

This experiment will consist of a sequence of 15 trading periods in which everyone in the room will have the opportunity to buy and sell in the same market. The currency used in the market is Francs (Fr), but the cash payment to you at the end of the experiment will be in pounds. The conversion rate is 360 Francs to 1 pound.

In each of the 15 periods, you will be able to buy and sell assets. Much like assets in a real stock market, these assets are simply an entitlement to certain revenue. In this game, each asset entitles you to receive 360 Francs (1 pound) at the end of the experiment. You will begin the game with 10 assets. During the game you may buy or sell your assets to other players. The amount you sell your assets for is completely up to you. The assets you own are carried over from one trading period to the next.

In order for you to buy assets you need cash. A 10,000 Franc loan will be provided to allow you to begin trading. This quantity will be subtracted from your final earnings at the end of the experiment.

At the end of each trading period you will receive either cash dividends or pay holding costs for each asset that you own. At the end of the experiment you will be paid a final dividend of 360 Francs (1 pound) for each asset that you own.

After trading has finished for each period, the computer will randomly determine the dividend or holding cost for that period. This will result in 1 of 4 possible outcomes, so that each asset you hold will either:

1) cost you a holding cost of 24 Francs
2) cost you a holding cost of 16 Francs
3) earn you a dividend of 4 Francs
4) earn you a dividend of 36 Francs

Each of these four possibilities is equally likely.

Note that the average dividend/holding cost in each period is 0. This can be shown to be the case over 4 periods:

Each of the 4 possible holding costs or dividends has a 25% chance of occurring. So over 4 periods one of the possibilities will occur once on average (4 x 25% = 1). For example, a possible sequence of holding costs and dividends might be:

Period 1) -24 Fr
Period 2) -16 Fr
Period 3) +4 Fr
Period 4) +36 Fr

So on average, the total of the dividends and costs for four turns will be (-24 -16 +4 +36 = 0), as each of the four possibilities will occur once on average.

This argument can be expanded to any number of periods, which means that, on average,

dividends and costs add up to zero. We also know that at the end of the experiment each asset that you own will pay you a one-off dividend of 360 Francs.

Therefore, this means that each asset has an average value of 360 Fr.

Trading Game: Trading Period

Trading time: At the beginning of each period you can buy and sell assets for a period of 2 minutes. A counter on the screen will show how much time is left to trade in each period.

Selling: You can offer to sell an asset by announcing to all the other people in the experiment that you are willing to sell an asset at a specific price, which you choose. Each person in the room will see this announcement, and anyone can choose to accept it or ignore it. If someone accepts it, then one asset is transferred to them, and the amount of Francs equal to the specified price is transferred to you. If nobody accepts your offer, then nothing happens.

Buying: You can offer to buy an asset by specifying a buying price. The procedure is then the same as the one for selling the asset: if someone agrees to sell at this price, then you will gain an asset at the price you offered. If no one agrees to sell at this price, then nothing happens.

Accepting offers: You can accept other players' offers by selecting them and pressing "Buy" or "Sell", depending on whether it is an offer to sell, or an offer to buy.

Below is a screenshot of a trading stage:

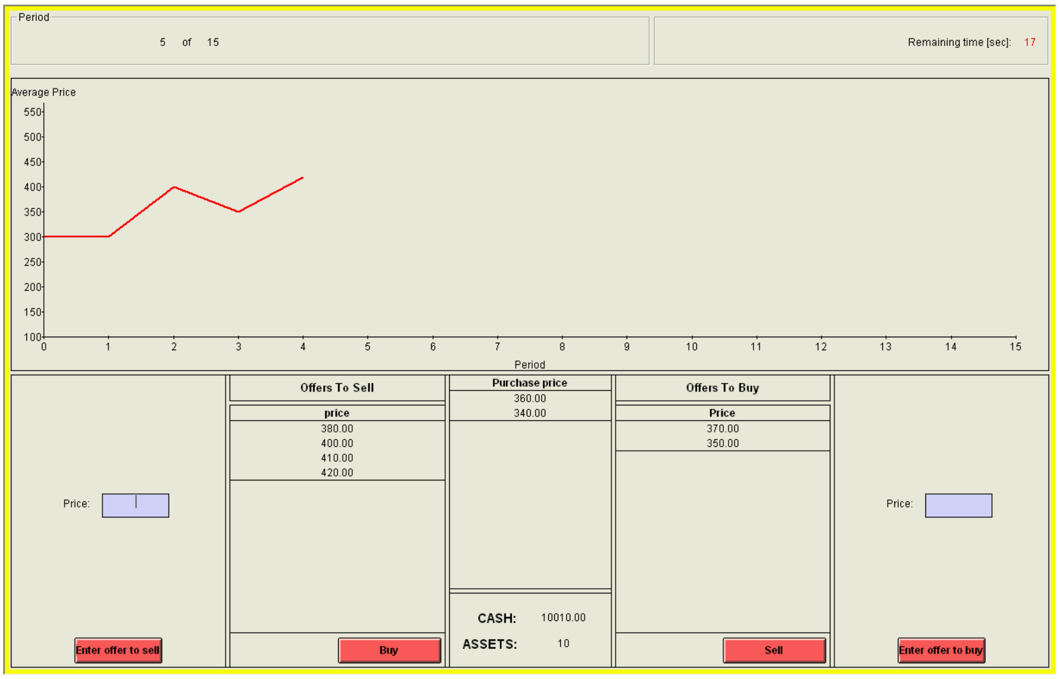

The top of the screen shows the trading period and the time remaining for trading in that period (in seconds).

In the upper half of the screen there is a graph displaying the history of prices for the asset in the previous periods. This is computed for each period as the average price at which assets have been bought or sold.

The bottom half of the screen contains five boxes:

1. Offering to sell: The first box from the left is where you would enter an **offer to sell** – the price at which you are offering to sell an asset to the other players. To do so, enter an amount in the small blue box and press "Enter offer to sell".

2. Buying: The second box from the left, **'Offers to Sell',** displays all the offers to sell made by players in the game. They are ordered so that the best offers come at the top. Hence, in this case, the best offer to sell corresponds with the cheapest price, which is 380Fr. To accept any offer you would click on one of these numbers (e.g. 380.00) and press **'Buy'**. In this case, that would mean that one asset is transferred to you, and 380Fr are transferred from your account to the seller's account.

3. Purchase prices: The box in the middle displays the **purchase prices**, that is, the prices at which other players have bought or sold assets in the current period. In this case, there have been two transactions: one for 360Fr and another for 340Fr.

4. Selling: The fourth box, **'Offers to Buy'**, displays all the offers to buy made by the players in the game, and works in a similar way to the second box. However, in this case, the best offers are the ones with the highest price, so these will be at the top. To accept an offer, select it and press **'Sell'.**

5. Offering to buy: The fifth box is where you can **enter offers to buy** – the price at which you are offering to buy an asset from the other players. This works in the same way as the offer to sell box.

Trading Game: Dividends and Holding costs

When trading time runs out at the end of each period, a screen will be displayed to indicate that the trading period is over.

The computer will then randomly determine the dividend or holding cost for that period. This will be the same for everyone.

For 30 seconds, the screen will display your balance of cash, the number of assets you hold, and the total dividends and holding costs up to and including that the current period.

At the bottom of the screen, you will be asked to enter a guess for the average transaction price of assets in the next period. **You will be paid 10 pence for every correct guess.** You must enter your guess within the 30 seconds limit.

At the end of the 15 periods the screen will show a final report of your balance of cash, assets, total dividends and holding costs.

Trading Game: Results

You will begin the experiment with 10 assets and an initial cash loan of 10,000 Fr.
Your final payoff will be the profit you make using your initial 10,000 Fr fund.

At the end of the experiment your earnings will be equal to:

- Final cash balance, minus the 10,000 Fr loan
- Final number of assets x 360 Fr
- plus sum of dividends during the experiment
- minus sum of holding costs during experiment

Remember that the conversion rate is 360 Francs to 1 pound, so each asset that you have at the end of the 15th period will pay you 1 pound.

Instructions: trading simulator

In this game you will be given a certain amount of money (£10.00) to invest.
You will be presented with a choice of two stocks, and you are free to choose how much of your money you wish to invest in these two options. The prices of the stocks will vary over the course of the experiment, and your aim is to maximise your profit.

The screen you see will look like this:

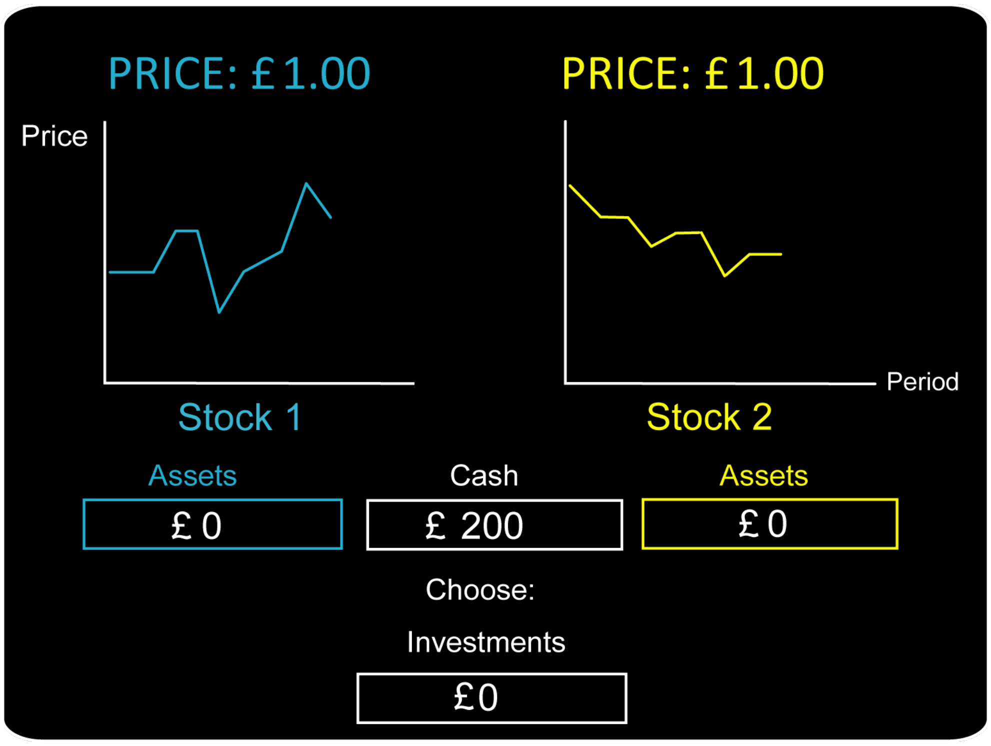

In each turn, you will be able to:

1) **Invest in each stock**
2) **Guess the next price change of each stock**

1. Choosing a Stock

At the start of each turn, you will be asked to **choose** which stock you wish to invest in first by **pressing 1 or 2**. You will always be able to invest in both stocks during a turn.

2. Investing in a stock: what are my options?

Once you have chosen which stock you want to invest in first, you can decide whether you want to buy or sell any of this stock.

At the beginning of each turn you will be able to buy or sell assets in both stocks, and at the end of the turn this investment will be automatically cashed in. Buying and short-selling are two ways of investing which offer alternatives depending on how you think the price of a stock will change.

Buying assets

When you invest you have the option of buying assets, which are then sold at the end of the turn. If you buy assets and the price **increases**, then when you sell the assets at the new higher price, you will make a profit.

When you buy assets [by entering a positive amount], the value of those assets is temporarily added in the assets box below the stock you invested in, and the amount of cash you have is reduced.

For example: if you bought £100 worth of assets at a price of £1 per asset, and the price increased to £1.10 (10% increase), your investment will increase to £110 at the end of the turn, giving a profit of £10.

Or, if the price went down to £0.90 (10% decrease), then your investment will decrease to £90 at the end of the turn, giving a £10 loss.

Selling assets

When you invest you also have the option of selling assets, which you have to buy back at the end of the turn. If you sell assets and the price **decreases**, then when you buy the assets back at the new lower price, you will make a profit (this is called short-selling).

When you sell assets [by entering a negative amount], the value of those assets is temporarily subtracted in the assets box below the stock you invested in, and the amount of cash you have is increased.

For example: If you sell £100 worth of assets at £1 per asset (100 assets in total), and the price decreased to £0.90, then you would be able to buy back 100 assets at the lower price, £0.90, and make a profit of £10.

Or, if the price were to increase to £1.10, you would have to buy back the assets at the new higher price, and you would make a loss of £10.

How much can I invest?
You begin the game with £10.00, which is represented by '1000' in the cash box.
The total value of your investments is represented in the box marked 'Investments'.
A) Buying in both stocks:
The maximum amount you can invest when buying is the amount of cash you have, the minimum investment is 0. Therefore you can invest any proportion of this in either stock, as long as the total does not exceed your cash.

B) Selling in both stocks:
The maximum amount you can invest when selling is equal to the amount of cash you have, the minimum is 0. Therefore you can invest any proportion of this in either stock, as long as the total does not exceed your cash.

C) Buying and Selling:
Buying and selling are opposite processes:

- When you buy stocks, <u>you get assets in advance</u> which you sell later.
- When you sell stocks, <u>you get cash in advance</u> and buy back assets later.

Therefore, if you want to buy more assets than the amount of cash you have, you can sell assets in the other stock to pay for the investment.

For example: You have 1000 cash, and you want to buy 1500 assets in Stock 1. If you sell 500 assets in stock 2, your cash will be 1500.

The maximum you can sell is the value of your cash. Therefore, if you have 1000 cash, and sell 1000, you will then have 2000 cash. The maximum buying investment then is 2000.

Your maximum investment is then 3000, or 3 x your current cash, and the minimum investment is 0.

Total Investments

The total value of your investments for each turn is shown in the box 'Investments'. The size of your investments will determine how much money you can potentially win or lose in each turn.

3. Guessing the next price

During each turn, you will also be asked to guess what the next price of each stock will be to the nearest penny. The accuracy of these guesses will influence the amount of money you receive at the end of the experiment. Your average accuracy as a percentage will be multiplied by £10 and this will be added to your final payoff. For example, if you guessed on average 70% (a 30% error), you would receive an additional £7.

<u>Order of questions in each turn:</u>

1) **Choose which stock to invest in first**
2) **Select amount to invest in 1st Stock**
3) **Select amount to invest in 2nd Stock**
4) **Guess the next price of 1st Stock**
5) **Guess the next price of 2nd Stock**

The prices of each stock will then update. If you bought assets, these will be sold at the new price. If you sold assets, these will now be bought back at the new price. If you made money on an investment the assets box will flash green, if you lost money it will flash red.

Stock Practice

Before you start trading you will be given a quick practice to make sure you understand how the game works. You will have 5 seconds to complete each decision, e.g. investment or guess, after which the computer will automatically move to the next stage.

Quiz

1) If the stock price was £1.00, and you invested £100, what would your profit be if the new price of the stock was £1.05?
2) If the stock price is £1.00, and you short-sell £100, what would your profit be if the price went down to £0.90?
3) If the stock price of both stocks is £1.00, and you invest £100 in each stock, what would your profit be if the new price for stock1 is £1.10, and the new price for stock2 is £0.90?
